# Polyurethane Nanocomposites with Open-Cell Structure Modified with Aluminosilicate Nano-Filler

**DOI:** 10.3390/ma17225641

**Published:** 2024-11-19

**Authors:** Joanna Paciorek-Sadowska, Marcin Borowicz, Janusz Datta, Łukasz Piszczyk, Paulina Kosmela, Iwona Zarzyka

**Affiliations:** 1 Department of Chemistry and Technology of Polyurethanes, Faculty of Materials Engineering, Kazimierz Wielki University, J. K. Chodkiewicza 30, 85-064 Bydgoszcz, Poland; m.borowicz@ukw.edu.pl; 2 Department of Polymer Technology, Gdańsk University of Technology, Narutowicza 11/12, 80-233 Gdańsk, Poland; janusz.datta@pg.edu.pl (J.D.); lukpiszc@pg.edu.pl (Ł.P.); paulina.kosmela@pg.edu.pl (P.K.); 3 Department of Chemistry, Rzeszow University of Technology, 35-959 Rzeszow, Poland; izarzyka@prz.edu.pl

**Keywords:** nano-filler, flexible polyurethane foams, polyurethane properties, halloysite

## Abstract

Nanocomposite flexible polyurethane foams (nFPUfs) were obtained by modifying the polyurethane formulation by adding a halloysite nano-filler in the amount of one to five parts by weight per hundred parts of used polyol (php). Flexible polyurethane (PU) foams with an open-cell structure and with a beneficial SAG factor were obtained. Premixes with nano-filler had a lower reactivity than the reference PU system. This favored the production of smaller cells, but with a more rounded shape in comparison with the REF foam without the nano-filler. During the study, the morphology and physical and mechanical properties were characterized, including apparent density, compressive stress, rebound flexibility, SAG factor, closed-cell content, and thermal stability, and compared with the properties of the unmodified reference foam. Scanning electron microscopy (SEM) showed that the cell structures of all prepared foams were open, and the cell size decreased with increasing nano-filler content. Apparent densities, SAG factors and rebound flexibilities of the foams increased with the increase of nano-filler content, while the resistance to permanent deformation showed the opposite trend. The proper selection of raw materials and optimally developed polyurethane formulations allow for obtaining environmentally friendly foams with favorable functional properties, taking into account price and the needs of sustainable development in the synthesis of flexible foams dedicated to the upholstery industry.

## 1. Introduction

Innovative polyurethane materials are modern materials designed to meet specific requirements, such as mechanical strength, durability, comfort or fire safety. They are developed using modern technologies and are used in various industries, e.g., furniture, civil engineering, automotive and packaging [1,2].

However, the use of such innovative materials is not without challenges. One of the biggest challenges is the cost of these materials, which can be much higher than in the traditional materials. This applies in particular to the most modern materials, the technology of which is based on new, non-standard solutions [3].

In polyurethane technology, a great deal of emphasis is placed on sustainable innovation in their development. Modern materials should not only be innovative and environmentally friendly but also economically viable.

With a view to innovation and significant investments in research and development, technology and environmental protection, the most important tasks for polyurethane plastics in terms of sustainable development are already being implemented. These include, among others, the use of recycled plastics and bio-based raw materials for the production of polyols, the use of innovative materials in final products, designing with recycling in mind, as well as the intensive development of innovative technologies, such as chemical recycling. These activities carried out in many centers allow the polyurethane industry to provide solutions that respond to the challenges of the modern world.

The market is dominated by polyurethane materials obtained from petrochemical raw materials. However, there is a noticeable trend of replacing them with products made from renewable raw materials [4,5]. The synthesis of polyurethanes from alternative “green” substrates, or the use of auxiliary substances in the form of processed plant materials (e.g., fruit pomace) or minerals (montmorillonite, halloysite), are no longer seen as unusual topics for research on polyurethane materials. The increasing demand for plastics, including those obtained by new methods and modified with environmentally friendly substances, indicates the need for their continuous development.

Polyurethane composite materials are currently one of the intensively developing and thoroughly studied groups of engineering materials, which is confirmed by the research results of, among others, polyurethane foams containing recycled fillers [6,7,8,9,10,11,12,13]. Most of the fillers used to obtain polyurethane foams require prior appropriate preparation. Very often, this is carried out in complicated and energy-consuming technological processes, which significantly increases the price of the final material [14,15,16].

The latest research directions aimed at improving the functional properties of polymeric materials include the possibility of using nano-additives, the particle size of which does not exceed 100 nm. The size of the contact surface of the polymer matrix and the filler has a significant influence on all properties of the materials. Changing the size from the micro- to the nano- scale increases the specific surface area of the added filler and allows the use of smaller amounts of filler to obtain the desired properties. Polymer nanocomposites exhibit better mechanical and barrier properties, higher fire resistance and thermal stability, and sometimes also lower smoke emission in comparison with unmodified materials [17,18,19]. It is important that the improvement of composite properties is visible even with a small addition of nano-filler, and its share is no more than 10 wt.%; usually it is less than 5 wt.% [20,21].

In recent years, attempts have been made to use various materials of mineral origin as reinforcement for polyurethanes. These include clay minerals from the kaolinite group due to their great prevalence in nature. These minerals are one of the main components of the Earth’s crust. Halloysite is also one of them. The combination of this type of reinforcement with a polyurethane matrix ensures obtaining an economically attractive product, and the use of an appropriately developed technology for its production guarantees favorable utility and operational properties. Such minerals can be successfully used to produce polyurethane composite materials used in various industries. Due to its specific structure, halloysite exhibits the characteristics of layered and tubular fillers.

**Figure 1:**
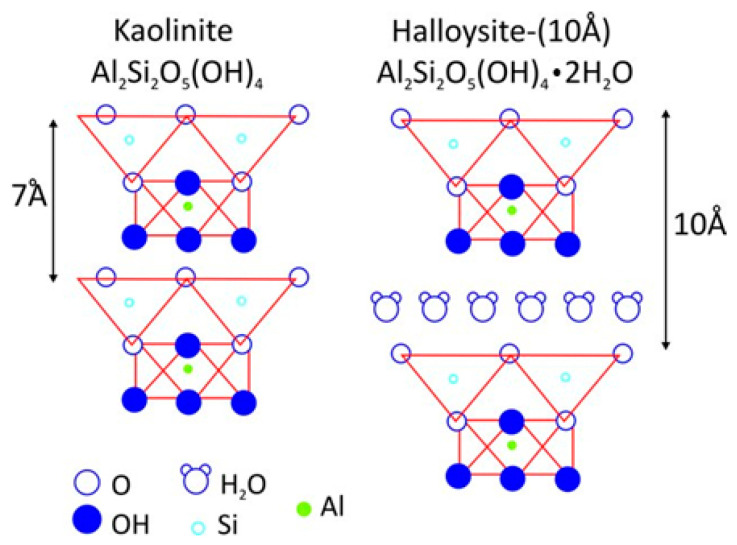
Crystal structure of halloysite with a marked monolayer of water molecules in the interlayer space [23].

The morphology of the halloysite grain depends on its origin and, above all, on the crystallization conditions in the geological environment. Halloysite is a naturally occurring nanoclay that is formed by weathering of aluminosilicates in an acidic environment, with the constant presence of water. As a result of this natural process, a monolayer of water molecules is formed in the interlayer space, which reduces the electrostatic interaction between octahedral aluminum layers (Al-OH) and tetrahedral silicon layers (Si-O-Si) (Figure 1). It is characterized by a porous spatial structure, which is formed by a layer of silicon tetrahedra and a layer of aluminum octahedra. The individual layers are separated by a free space, which can contain absorbed ions and molecules (including water), which are loosely bound to the surface of the plates, most often by hydrogen bonds. About 30% of this mineral is made up of rigid, straight nanotubes with a length of about 2 µm and a diameter of 10–150 nm. It can be a cheaper and more ecological alternative to expensive carbon nanotubes [22].

Halloysite can occur in two forms:

- hydrated (10 Å), containing four H_2_O molecules forming a monomolecular layer in the interlayer space with the chemical formula Al_2_Si_2_O_5_(OH)_4_·nH_2_O;
- dehydrated (7 Å) in the chemical formula Al_2_Si_2_O_5_(OH)_4_, also called metahaloysite.

In the hydrated form of halloysite, the distance between the planes is 10.1 Å. In the dehydrated form, this distance is 7.2 Å. The difference between these distances is equal to the thickness of the monomolecular layer of the water molecule (2.9 Å). Hydrated halloysite very easily loses the water contained between the tetrahedral and octahedral layers.

However, regardless of the type of used nano-filler and its form, making a good quality nanocomposite of flexible polyurethane foam dedicated to the upholstery industry is a very difficult task. The significantly higher value of free surface energy of nano-fillers than polymers is such that fine filler particles tend to agglomerate. In addition, the lack of active groups on the surface of the nano-filler means that the possibilities of creating bonds or interactions at the polymer–filler boundary are severely limited. As is known, the degree of dispersion of the nano-filler in the matrix depends on the type of polymer and its ability to wet the mineral filler. In general, the macroscopic properties of composite materials depend on the morphology of the phases present in the system and their stabilization. Therefore, to achieve the best possible properties of the final product, appropriate adhesion forces between the matrix and the nano-filler are necessary. To achieve the expected properties of nanocomposites, a properly developed formulation and method of producing the material should be used.

The use of different fillers to modify flexible foams usually causes deterioration of functional properties, such as a rapid increase in CLD_40_ hardness, a decrease in rebound flexibility or a decrease in the SAG factor. Flexible foam manufacturers struggle with this problem, and many research centers conducting research in this area identify it. The use of cheap filler could have a twofold application. First, the use of cheap fillers can have a positive effect on reducing the price of the finished product, which is not the cheapest. Secondly, due to the specific properties of the filler, it was assumed that the basic performance parameters of the obtained foams would be improved, including the rebound elasticity or SAG factor. Previously conducted research on the use of plant bio-fillers has shown that their addition into the polyurethane matrix results in a decrease of functional properties [24]. Therefore, considering the need to develop more sustainable polyurethanes dedicated to the upholstery industry, the aim of this research was to obtain cheap nanocomposite flexible polyurethane foams with improved thermal and mechanical properties. For this purpose, the research was aimed at obtaining polyurethane foams using unmodified natural mineral halloysite as a nano-filler. The low price of halloysite nanoclay combined with its specific properties would lead a foam polymer/nanoclay with improved performance properties, including the rebound elasticity and SAG factor.

## 2. Materials and Methods

### 2.1. Materials

The selection of appropriate raw materials and the best technique for preparing nanocomposite flexible polyurethane foams (nFPUfs) is crucial to obtain the desired properties of a specific material. Suitable raw materials for the development of the polyurethane formulation were selected based on their properties, purpose and influence on the formation of a polyurethane matrix containing nano-filler.

**Figure 2:**
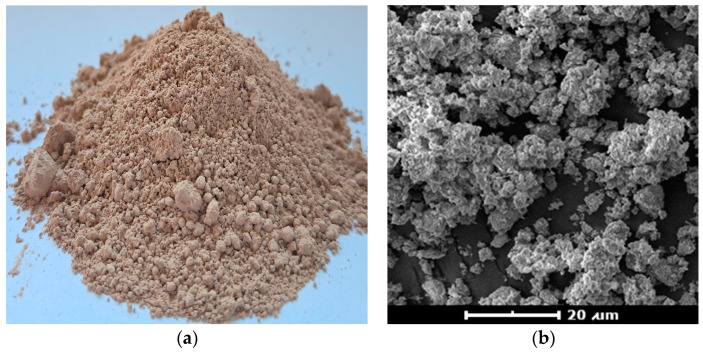
(**a**) Halloysite clay from the Dunino mine; (**b**) halloysite clay from the Dunino mine, magnification 4100×.

Rokopol F3600 (PCC Rokita S.A., Brzeg Dolny, Poland) polyether polyol was used to synthesize nFPUfs. It is a copolymer of ethylene oxide (EO) and propylene oxide (PO) based on glycerine as a starter. It is a homogeneous, clear liquid, with an OH number of 48 mg KOH/g. Ongronat 1080 (mixture of 2,4- and 2,6-TDI isomers) was used as the isocyanate raw material. It has a 2,4- isomer content of 81%. It was purchased from BorsodChem (Kazincbarcika, Hungary). The additives used were Tegostab BF2370 as a silicone-based surfactant; DABCO BL-11 as a catalyst of hydrolysis of NCO groups; DABCO 33LV as a catalyst of polyol and isocyanate reactions; and KOSMOS T9—tin octanoate—activating the gelation reaction. The supplier of the used additives was Evonik Industries AG (Essen, Germany). Carbon dioxide (CO_2_) produced in reaction of NCO groups with distilled water was used as a chemical blowing agent. Halloysite was used as a physical nano-filler for flexible PU foams. It was supplied by the halloysite mine Dunino Sp. z o.o. (Toszek, Poland). The Dunino mine is one of three halloysite mines in the world (in addition to it, there is one mine in New Zealand and one in the U.S.A.). The Dunino mine has resources containing at least 10–12 million tons of halloysite. Dunino is an open-pit deposit with a 0.5 ÷ 1 m thick overburden, which has a significant impact on the extraction costs, which are not high. The thickness of the halloysite layer is up to 20 m. The entire deposit is characterized by a homogeneity of composition and high purity. Halloysite from the Dunino mine is characterized by a large specific surface area (approx. 65,000–400,000 m^2^/kg) and high porosity (60–70%). The obtained halloysite contains about 39.5% Al_2_O_3_ and 46.55% SiO_2_, which is 20.9% aluminum and 21.76% silicon in terms of pure elements. The rest are impurities, which include compounds of iron, titanium, calcium and sodium. The physical form of the mined halloysite is shown in Figure 2a,b. The chemical composition of the nano-filler is presented in Table 1 (data according to the manufacturer, Dunino Sp. z o.o., Toszek, Poland).

### 2.2. Obtaining nFPUfs

The structure of PU foams and their properties are the result of several processes occurring simultaneously during the foaming process. In turn, the variety of applications of flexible PU foams is the result of the possibility of controlling their properties to a wide extent, both by changing the type and quantity of raw materials in the reaction mixture.

In the process of obtaining flexible foams, several stages can be distinguished. They are characterized by determining parameters such as dielectric polarization, rise rate, reaction time and reaction temperature. The values of these parameters have a decisive influence on the formation of the individual elements that make up the polyurethane structure.

A one-step method was used to produce the foams in this research. This method involves two main components: A (polyol + additives) and B (isocyanate). During the laboratory work, component A and component B were weighed in separate half-liter polypropylene cups. The appropriate amount of polyol and all additives (surfactant, catalysts, blowing agent and nano-filler) were weighed into the cup of component A according to the formulation given in Table 2. Only Ongronat 1080 was weighed into the cup of component B. The applied method enabled the optimal preparation of the polyol premix. The preparation consisted of the precise weighing and then perfect mixing of the components that do not react with each other. This enabled a short contact of the reacting components in the next stage, ensuring the correct course of the appropriate reactions.

During this research, six flexible polyurethane foams were obtained: one reference foam (REF) and five foams modified with halloysite (H1–H5). The amount of nano-filler in the individual foams increased from 1 php to 5 php (by 1 php in each subsequent foam). Formulation of the refence foam is presented in Table 2.

After pouring the contents of the cup with component B into component A, whole mixture was quickly and thoroughly mixed using a high-speed mixer equipped with a dispersion tip (mixing parameters: 10 s, 1800 rpm, mixer length 20 cm). After that, the whole mixture was poured into a heated rectangular mold with dimensions of 25 cm × 25 cm × 30 cm, where it rose freely. The mold temperature was 120 °C and was maintained until the foam was removed from it. During the foam rise, many phenomena occur, including physical phenomena such as cell formation, color change, viscosity increase, cell opening and structure stabilization; and kinetic phenomena such as the hydrolysis reaction of isocyanate raw materials or the reaction between polyol and isocyanate raw materials. Then, the molecular weight also increases. All these phenomena have a significant effect on the formation of the open-cell structure of flexible polyurethane foam.

The foam rise time was observed. Particular attention was paid to the timing of rise termination. After the foam rise stage, the foam stabilization stage begins, during which further chain extension reactions and cross-linking of the PU occur. At the end of each foam rise, a “squat” of the foam was noticeable. This is a typical phenomenon for open-cell foams. It consists of a cracking of the walls combined with the release of gas present in the cells. Meanwhile, the viscous mixture transforms into a solid high molecular weight polymer, and its surface becomes dry [25].

After the foam had grown in the mold and the parameters accompanying the foaming process had been measured, the foam was placed in an air dryer with forced circulation at 120 °C for 2 min. Foam after removing from the dryer was subjected to organoleptic evaluation. Obtained foams ware subjected to 72 h seasoning after positive organoleptic evaluation. The foaming process was repeated three times for each foam formulation. The obtained flexible foams, after seasoning, were cut into appropriate samples and subjected to tests.

### 2.3. Methods

The addition of nano-fillers to a PU premix changes the course of the polyol and isocyanate reaction. Moreover, their presence can cause problems at every step of the foaming process, which is related to, among others, the increase in the mixture viscosity, difficulties with homogenization and the cell formation process, and above all, the change in the kinetics of the polymerization reaction [26,27].

The foaming process of the nFPUfs was analyzed by a FOAMAT device with specialist software (Format-Messtechnik, Karlsruhe, Germany). The apparatus enables repeatable measurements of rate of foam rise, dielectric polarization of the whole mixture and temperature of foaming process. After synthesizing the flexible polyurethane foams, they were subjected to visual evaluation, assessing the condition of the foam surface, possible shrinkage and discoloration.

The microstructure of each sample was measured using a SEM Hitachi SU8010 (Hitachi High-Technologies Co., Tokyo, Japan) microscope. Measurement of the foam structure was made at an accelerating voltage of 10 kV. The working distance used in this test was 10 mm, and the magnification was 130×. The use of SEM microscopy for sample analysis allowed for the estimation and statistical analysis of the number, shape and dimensions of cells and their degree of openness.

The characteristic functional groups in the organic matrix of nFPUfs was analyzed using Fourier transform infrared spectroscopy (FTIR). All spectra were obtained using a Nicolet iS20 spectrophotometer (Thermo Fisher Scientific, Waltham, MA, USA) in the range from 400 to 4000 cm^−1^.

Apparent density of nFPUfs was determined in accordance with PN-EN ISO 845:2010 [28] from a sample dried for 2 h at 40 °C. Apparent density (*AP*) is defined by the equation

$$AP=\frac{m}{V}$$

where

*m* is material mass (kg), and
*V* is the total volume of sample (m^3^).

The CLD_40_ hardness was determined in accordance with PN-EN ISO 3386-1:2000 [29] using a Zwick/Roell Z005 (Zwick Roell Group, Ulm, Germany) universal testing machine. CLD_40_ is the force acting on the sample required to compress the foam by 40% of its initial height. This measurement expresses the load-bearing capacity of the foam.

The determination of rebound flexibility was performed in accordance with DIN 53573 [30] by the Schöb elastomer, device type 5109 (Gibitre Instruments srl, Bergamo, Italy). A pendulum with a potential energy of 0.196 J was used for the tests, with a weight of 101 g. A sample of foam with dimensions 80 mm × 80 mm × 50 mm was placed in an apparatus holder and hit by a weight attached to a pendulum. The rebound flexibility was determined based on the return deflection of the pendulum after hitting the sample. The assay result was the mean of at least five measurements of the one type of sample.

The SAG factor was determined in accordance with ISO 3386-1 [28]. The equation was used to calculate the parameter.

$$SAG\;factor=\frac{{CLD}_{65}}{{CLD}_{25}}$$

where

*CLD*_65_ is the pressure required for compressing the foam by 65% (kPa), and
*CLD*_25_ is the pressure required for compressing the foam by 25% of the initial height (kPa).

The permanent deformation test principle consists of holding the tested foam for a specified time, at a specified temperature. Measurement was carried out under a constant load and recording the sample thickness before testing and after removing the load. The test involved compression of foams to 50% of the initial height at a temperature of 60 °C for 22 h. According to the standard, such parameters best simulate the permanent deformation that occurs as a result of the long-term use of foam, among others in the furniture industry.

Closed- and open-cell content of REF. Foams and nFPUfs were measured by an Ultrapyc 5000 gas pycnometer (Anton Paar, Graz, Austria). All foams were analyzed in an inert atmosphere (nitrogen). The pressure of the used gas was 3.0 psi, and the measurement temperature was 20 °C. All samples were tested using a 45 cm^3^ cell size.

The thermomechanical properties of the obtained foams were tested by dynamic mechanical analysis (DMA). For this purpose, a Q800 DMA apparatus (TA Instruments, New Castle, DE, USA) was used. All analyzed foams were cuboid samples with dimensions of about 17 mm × 12 mm × 4 mm. All samples were tested in single cantilever mode. The temperature range of measurement was from −130 to 100 °C. The heating rate was 4 °C/min.

The thermal resistance of obtained nFPUfs was evaluated by thermogravimetric analysis (TGA). This analysis was carried out by a Netzsch TG 209F3 apparatus (Netzsch, Selb, Germany). All foam samples were heated with a heating rate of 10 °C/min under an inert (nitrogen) atmosphere from 30 to 800 °C. The foams’ masses were about 10 mg.

## 3. Results and Discussion

### 3.1. Foaming Process of nFPUfs

**Figure 3:**
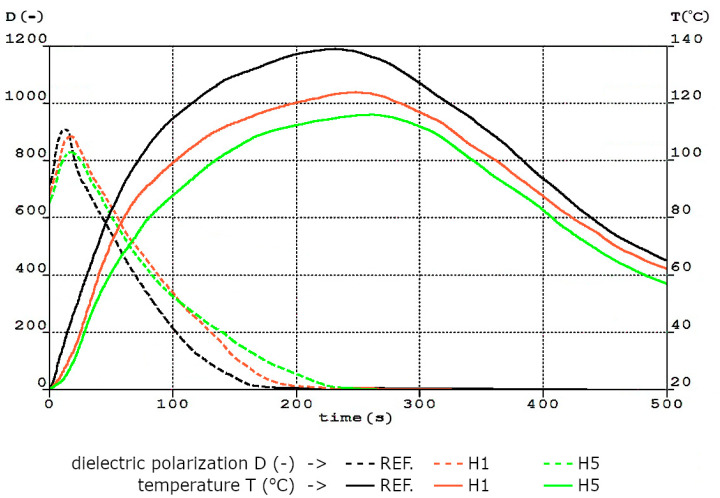
Dependence of temperature (T) and dielectric polarization (D) on reaction time for REF, H1 and H5 foams.

In the structure of flexible open-cell polyurethane foam, each cell is defined by the number of ribs, in the shape of convex polyhedrons with internally connected cells, creating a three-dimensional network of ribs and walls. Depending on the selection of raw materials in the formulation and foaming method used, the size, shape and number of cells, as well as the wall thickness and ribs, can be very diverse. In turn, the properties of the structure significantly determine the quality of the obtained polyurethane foam [31]. The foaming process course of the obtained foams is related to changes in characteristic parameters such as dielectric polarization, foam rise time and reaction temperature. The dependence between these parameters for selected foams is shown in Figure 3. Time 0 s was assumed to be the moment of pouring component B into component A. In turn, the temperature of the foaming process was measured by a thermocouple placed in the reaction mixture inside the apparatus mold.

A polyurethane system modified with a non-reactive physical filler is usually characterized by reduced reactivity in comparison with systems not modified physically. This is associated with a number of technological problems that can disrupt both the nucleation process and the growth and development of cells [32,33]. Therefore, this should be taken into account already at the step of developing the polyurethane formulation. A properly selected composition of raw materials and their proportions will counteract these negative effects. When halloysite was added to the system, changes in the parameters accompanying foaming were observed. An increase in the mixture viscosity was noted after mixing the nano-modifier in the polyol premixes. It simultaneously resulted in a decrease in the reactivity of the whole system. This is normal when using a physical modifier and has been observed in many scientific studies [34,35].

Obtained results indicate that the reference foam was characterized by a higher reactivity associated with the hydroxyl and isocyanate groups in the reaction mixture, and its changes can be observed based on the course of the D-curve. The formation of the polyurethane matrix leads to a gradual limitation of the mobility of these groups as the reaction progresses. Hence, a decrease in the dielectric polarization value is observed. A relatively intensive decrease in dielectric polarization occurs for the reference foam, which also confirms the fastest course of its foaming reaction. From the moment the test began, it reached a value of 0 after 180 s. After this time, the intensive foaming reactions are completed. This moment is illustrated in the T-curve and D-curve. The inflection point of the T-curve and a rapid decrease in the curve of dielectric polarization was associated with this. Then comes the gelation stage, which lasts much longer. The D- and T-curves then flatten out. The D-curves of the foams with the lowest and highest nano-filler content have a similar course but are shifted towards longer times. H1 (the foam with the lowest content of halloysite) reached a value of 0 after 219 s, while H5 (the foam with the highest content of halloysite) took 234 s. The higher reactivity of the reference system caused a shift in the curves, which was caused by the higher mobility of this system in comparison with the systems modified by halloysite. In addition, the modified foams had lower initial dielectric polarization values, which was the result of increasing the viscosity of the modified systems. The maximum core temperature of the REF foam was higher than that of the modified foams and reached 139 °C in 229 s. Based on the course of temperature changes, it can be concluded that the systems modified with the nano-filler were characterized by a lower reactivity than the REF system. A decrease in the core temperature and an extension of the time taken to reach it for the obtained H1 and H5 foams were observed, to 125 °C in 241 s and 114 °C in 257 s, respectively. Introduction of nano-filler into the PU matrix caused a slowdown in the foaming of the polyurethane systems. The process was disturbed by the higher viscosity of the systems, which affected the reduction of dielectric polarization and the decreasing of the temperature.

### 3.2. FTIR Analysis and Structure of nFPUfs

**Figure 4:**
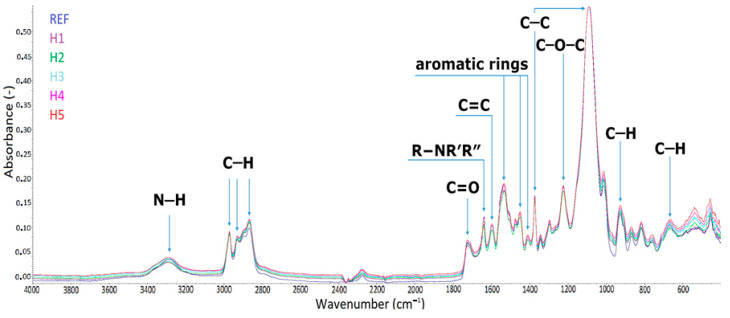
FTIR spectra of REF and halloysite-modified foams (H1–H5).

The FTIR method was used to analyze the presence of the characteristic chemical groups of the obtained flexible polyurethane foams. The obtained spectra are presented in Figure 4.

Characteristic functional groups for flexible PU foams were analyzed by the FTIR spectra. A detailed discussion of the bands is presented in Table 3.

**Figure 5:**
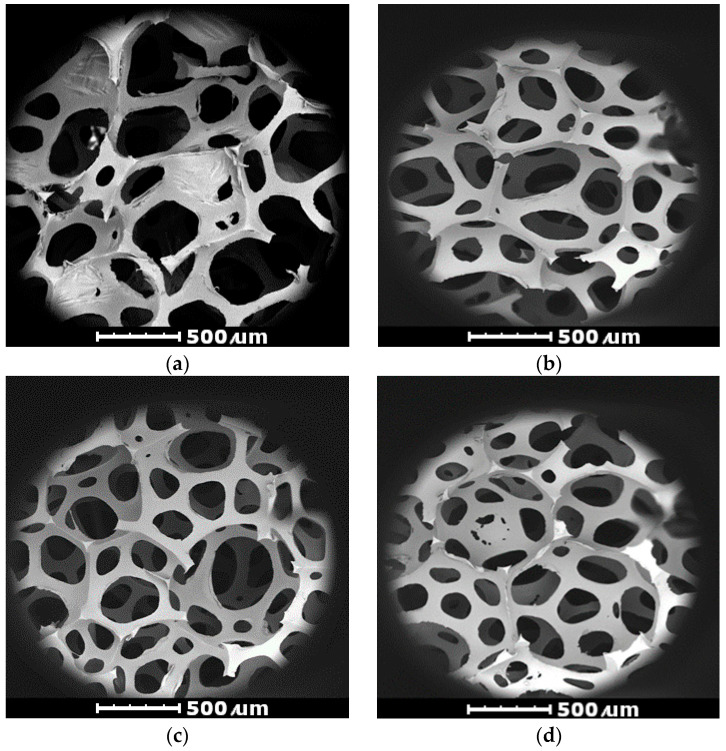
SEM micrographs of the cellular structure of the reference foam REF (**a**) and the foams modified with nano-filler, H1 (**b**), H3 (**c**) and H5 (**d**); magnification 130×, accelerating voltage 10 kV.

The development of cell structures during the foaming processes of nanocomposite foams is closely related to the physical properties of PU foams. Cells are remnants of gas bubbles formed in the first stage of the foam synthesis process. The process of foam structure forming consists of several stages, i.e., the nucleation process, growth and the stabilization of single cells in the created polyurethane matrix. Cell nucleation is the source of gas bubbles, which in nanocomposite polyurethane foams is carbon dioxide. The amount and size of the resulting gas bubbles are important factors, determining the properties of the obtained materials. The process of gas bubble nucleation in the conducted research was modified by using halloysite, which acted as heteronuclei. Due to the heteronucleation process leading to the formation of gas bubbles, the structures of H1, H3 and H5 foams with reduced cells and homogeneous size were obtained. Figure 5a–d shows the cellular structure of flexible polyurethane foams made using the SEM technique. The foam cells have a polyhedral shape, mostly consisting of open cells. The SEM micrograph of the reference foam showed a slightly deformed structure, which confirmed the collapse of some cells. This was the result of a much lower viscosity of the reference foam premix. The lower viscosity of the polyurethane system causes a rapid growth of the raw material mixture without a sufficiently rapid cross-linking of the structure [36]. In such case, the cells that are being formed also merge. This could be observed during this study. Fundamental differences were observed in the structure of the prepared foams due to the incorporated nanoclay. This was confirmed by the many small cells, resulting in foams with improved properties. The reference unmodified foams showed a much weaker structure than was the case for the modified foams. This was associated with the cracking of the matrix walls and ribs, which led to cell fusion. The residue of this cracking was observed as a deformed structure seen in the SEM micrograph of the reference foam (Figure 5a).

The nFPUfs had a uniform cell size distribution; the cellular structure was not collapsed or damaged. The increase in the viscosity of the reaction mixture caused by the presence of the nano-filler during the rise of the bubbles promoted the formation of a heterogeneous structure [37]. Changing the nucleation technique from homogeneous to heterogeneous and reducing the nucleation energy favored the formation of small, rounded cells that did not show a tendency to coalesce gas bubbles [38]. In the case of the analyzed flexible foams, the influence of cell anisotropy was not taken into account, because it has a negligible effect on the properties of the tested materials [39].

The proportion between closed and opened cells is major parameter of the foam’s structure. It is related in flexible polyurethane foams to properties such as thermal or acoustic insulation or water absorption. It could be seen that regardless of the analyzed foam series, the open-cell content was slightly increased by increasing the halloysite content in the modified formulations. Overall, the conclusion is that the foam has an advanced open-celled polyurethane matrix. The quantitative results of open-cell content are presented In Table 4. All obtained nFPUfs showed an increase in open-cell content in comparison with the reference foam. These effects can be clarified by the incorporation of nano-filler into the polyurethane walls, which resulted in greater cell opening [40,41].

### 3.3. Physico-Mechanical Properties of nFPUfs

A flexible polyurethane foam must have a high content of open cells to ensure good mechanical properties of products obtained from it [18]. The formation of cells and their rupture are the result of many physical phenomena and chemical reactions occurring throughout the technological process. Determining the balance point between these key phenomena is a very difficult task due to the large number of process variables. They concern, among others, the composition of the formulation, the mixing technique, the foaming method and the selection of the catalytic system. The right combination of the above factors at the right moment of the process leads to obtaining a product with optimal properties. If there is a sufficient number of open cells characterized by a stable structure in foams, this improves their functional properties. The addition of nano-fillers to the polyurethane formulation required a detailed analysis of the obtained results. The apparent density of systems with physical fillers may increase as a result of the increase in the composite viscosity during the foaming stage, which has been observed in many research centers [42,43,44]. The most common cause of this is the agglomeration of the filler into larger clusters and its uneven distribution in the polyurethane matrix. This causes a number of difficulties in characterizing the functional properties of non-homogeneous samples. Hence, examining the apparent density of samples from different places in the same material confirmed that the selected mixing method was effective and allowed us to obtain a well-homogenized nanocomposite. It is assumed that the optimum apparent density of a conventional flexible foam of medium density is in the range of 22–40 kg/m^3^. This is a foam with good compression resistance that is also economically viable. In addition, such foams are characterized by high durability and very high comfort of use, which is very important in the upholstery industry.

**Figure 6:**
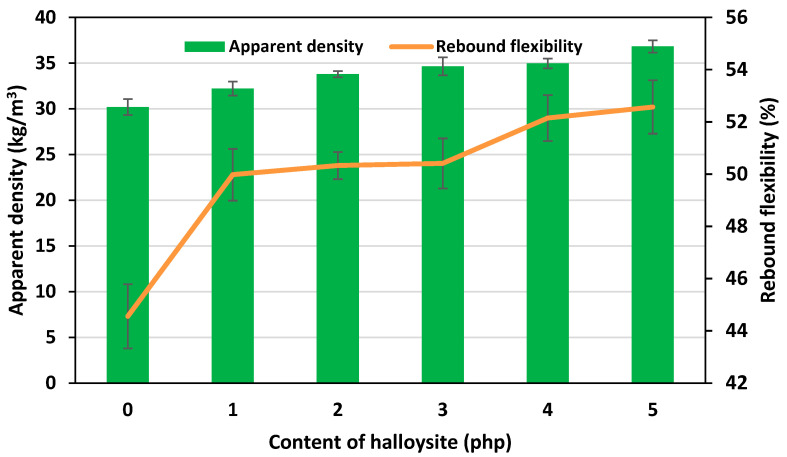
Relationship of the apparent density, rebound flexibility and the content of halloysite in nFPUfs.

The introduction of halloysite to the PU formulation contributed to an increase in the apparent density of the foams from 30.2 kg/m^3^ for the REF foam to 36.8 kg/m^3^ for the H5 foam, containing 5 php of filler. It was noted that the apparent densities of the produced foams were within the range of values of classic flexible polyurethane foams for many applications. Very similar results were obtained from several repetitions for each foam, which confirmed the homogeneous dispersion of the nano-filler in the polyurethane matrix. Obtaining nFPUfs with the desired apparent density was also made possible by the selection of a chemical blowing agent (carbon dioxide) for the polyurethane system. Typically, the so-called “light foams” (foams with lower apparent density) are obtained using a physical blowing agent which, by evaporating, additionally reduces the temperature of the foam block during the foaming process. However, such foams have a much lower mechanical strength and do not provide as much comfort of use as foams with a medium apparent density. The apparent density of flexible PU foams regards their rebound flexibility. The graph of the dependence between rebound flexibility and apparent density of the obtained foams is shown in Figure 6. It was noted that with the increase in the apparent density of flexible polyurethane foams, their rebound flexibility increased. As a rule, the use of physical fillers causes disruptions in the polyurethane structure. The added particles can agglomerate, which contributes to their uneven distribution, the formation of defects in the matrix and the deterioration of functional properties. In the case of the applied nano-filler, nanometric particles were introduced into the polyurethane structure, which did not cause defects in the matrix. Tests of samples taken from different locations of the obtained foam block did not show any differences in the values of the tested parameters. Moreover, the halloysite used as a modifier did not cause hardening of the polyurethane structure, and its even distribution, in addition to the ordering of the nFPUf structure, contributed to the increase in rebound flexibility despite the increase in apparent density. The lowest value of this parameter was found in the REF foam, which was characterized by the lowest apparent density. It was 44.56%. The highest rebound flexibility was characterized by the H5 foam, with the highest apparent density value, simultaneously containing the largest amount of filler. The value of this parameter was 52.57%.

**Figure 7:**
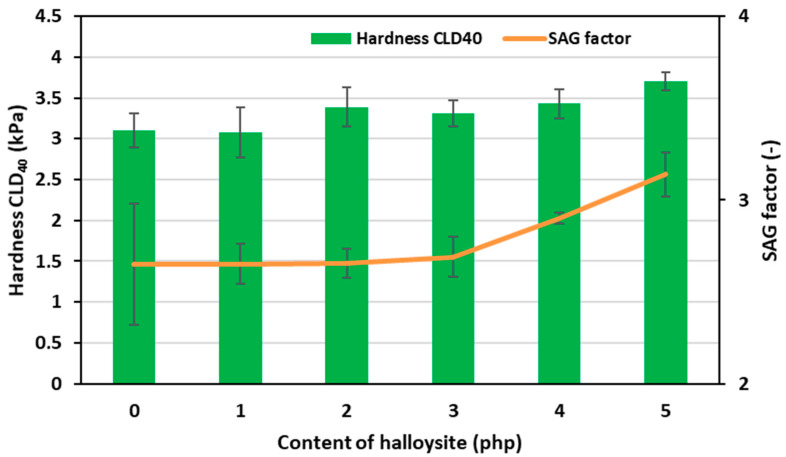
Relationship of CLD_40_ hardness, SAG factor and the content of halloysite in nFPUfs.

The CLD_40_ hardness values of the obtained flexible polyurethane foam nanocomposites containing halloysite and the dependence of SAG (comfort) factor on it are presented in Figure 7.

The CLD_40_ hardness of the obtained nFPUfs varied depending on the nano-filler content, ranging from 3.1 kPa to 3.7 kPa, and increased with the increasing apparent density of the obtained materials. The use of nano-filler in the formulation resulted in a strengthening of the polyurethane matrix and increased hardness of the foams. In turn, the SAG factor is usually increased by adding a physical filler. Typically, polyurethane formulations are designed to slightly reduce the force required to deform the foam by 25%, with a small increase in apparent density [44]. The even dispersion of halloysite in the polyurethane structure allowed the adjustment of the above parameters to meet this industrial requirement. The SAG factor of the obtained nFPUfs was in the range of 2.15 to 2.68. This was the optimal value for materials intended for the production of upholstery products. Ultimately, choosing a comfortable mattress or piece of furniture is a subjective matter and always depends on individual preferences. These results show better improved performance properties than those obtained in a previously published study on agricultural industry waste [24]. This is mainly attributed to the specific properties of halloysite nanoclay.

Elongation at the break and tensile strength of flexible PU foams are important properties related to the structure of the foam [45,46]. The yield stress is the minimum stress at which a material loses its elastic properties. The tensile strength is the maximum stress a material can withstand before it breaks. The relative elongation at break is the deformation of the sample at the moment of its break.

**Figure 8:**
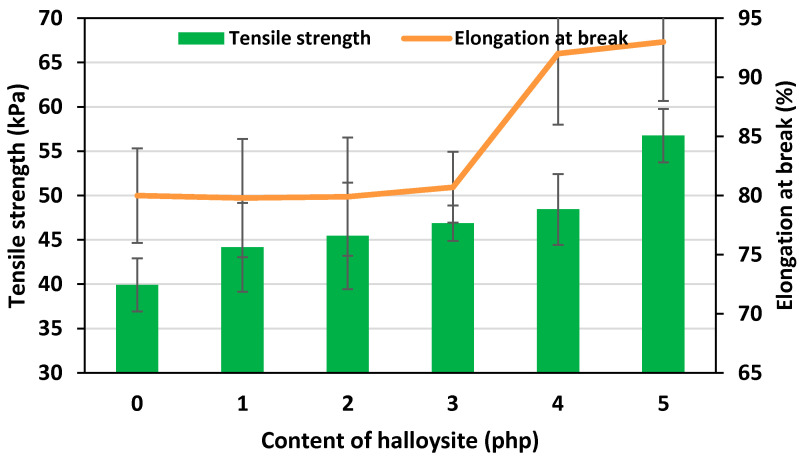
Relationship of the tensile strength, elongation at break and the content of halloysite in nFPUfs.

Elongation at break and also tensile strength are parameters that depend significantly on the PU matrix structure. Figure 8 presents the results of these parameters for the reference (unmodified) foam and foams containing increasing halloysite content.

The addition of halloysite increased the tensile strength and yield stress of nFPUfs (Figure 8). For the REF foam, it was 39.93 kPa, while for the foam with the highest content of halloysite, it was 56.76 kPa. Increasing the nano-filler content resulted in strengthening the walls of the PU matrix, and slower gelation allowed obtaining a regular structure with evenly distributed particles. The homogeneous distribution of the nano-modifier in the polyurethane matrix contributed to the reinforcement of the obtained material, which was demonstrated by the results of tensile tests. The observed increase in tensile strength also resulted in phase separation between the hard and soft matrix segments, which was caused by the presence of nano-filler [47,48,49].

**Figure 9:**
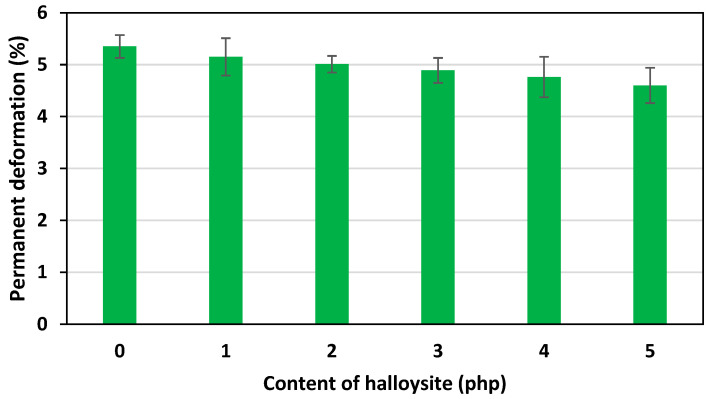
Dependence of permanent deformation on the content of halloysite in nFPUfs.

Addition of filler to the matrix of flexible PU foams contributed to a favorable reduction in permanent deformations compared to the unmodified foam. The relationship between permanent deformation and nano-filler content is shown in Figure 9.

As a result of the conducted tests, a decrease in permanent deformation was noted with the increase in the content of halloysite. nFPUfs were characterized by lower permanent deformation (4.60% for foam with the highest halloysite content) in comparison with the reference foam (5.35%). It was noticed that the higher the elasticity of the foams, the smaller the permanent deformations of the samples. The decrease in the permanent deformation values could be the result of the observed decrease in the reaction temperature during the foaming process of foams with the addition of a physical modifier. It was noted that the addition of halloysite slowed down the reaction rate between the hydroxyl and isocyanate groups. This caused the formation of more urethane groups in the rigid segments during the foaming process.

### 3.4. Thermal Properties of nFPUfs

**Figure 10:**
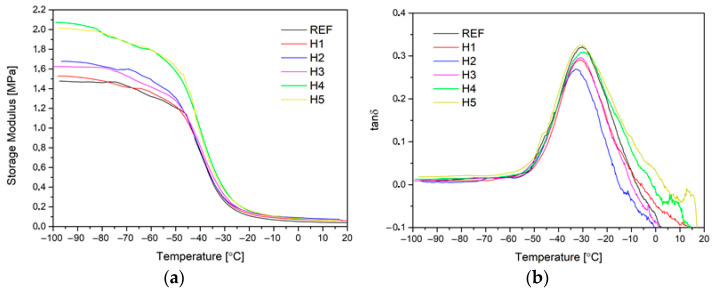
Temperature plots of storage modulus (**a**) and tan δ (**b**).

The dynamic analysis of the mechanical properties provided information on, among other things, the glass transition temperature (T_g_), the damping ratio (tan δ) and the effect of the filler on the stiffness of the flexible foams. The results are shown in Figure 10 (and Table 5). From the results obtained, it can be noted that the addition of halloysite filler does not significantly affect the value of the glass transition temperature, as read from the maximum of the tan δ peak. In all the samples tested, T_g_ ranges from −30 °C to −33 °C, and is characteristic of flexible PU foams [50]. Analyzing the values of the damping coefficient (tan δ), it can be concluded that all the tested samples show good damping capacity, as the maximum tan δ value is in the range of 0.27–0.32. The higher the tan δ value, the better the material damps vibrations [51]. No close correlation was observed between the amount of halloysite filler and the value of the damping ratio. It can be concluded that the addition of halloysite filler does not reduce the ability of flexible polyurethane foams to dissipate mechanical energy, which may indicate a lack of filler/polymer matrix interaction at the interface [52]. A significant effect of filler amount was observed by analyzing the values of the conservative modulus. As the amount of filler halloysite increased, the modulus values increased. This demonstrates the effect of filler on the stiffness of flexible PU foams. Similar relationships were observed in other works [53]. For negative temperatures, an increase of approx. 40% in the conservative modulus was observed for samples containing halloysite filler, compared to the reference samples H4 and H5, containing 4 and 5 php of filler.

**Figure 11:**
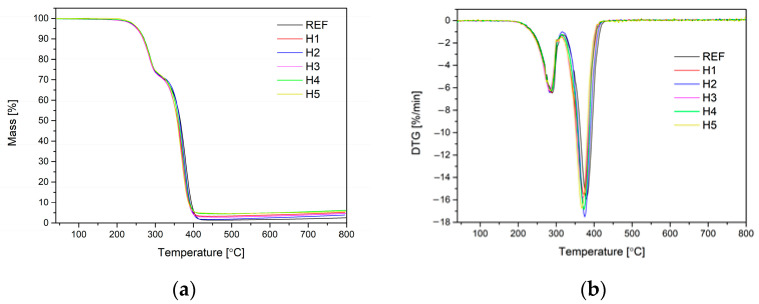
TG (**a**) and DTG (**b**) curves of the REF foam and foams with increasing content of halloysite.

Figure 11 shows the TG and DTG curves of the reference foam and polyurethane nanocomposites. Table 6 shows the results of thermal analysis. During the thermogravimetric analysis, the following parameters were determined: T_5%_—temperature of 5% sample weight loss; T_10%_—temperature of 10% sample weight loss; T_50%_—temperature of half the sample weight loss, the highest rate of weight loss during the test and the amount of the sample remaining after the test (residue).

The analysis of TG and DTG thermograms showed that the thermal decomposition of polyurethane nano-composites and the reference foam was basically identical in each case. Thermograms of the REF foam and H-series foams did not show any significant differences. This was evidenced by the results of characteristic temperatures, such as T_5%_, T_10%_ and T_50%_, which were each at a similar level of approx. 254 °C, 270 °C and 360 °C, respectively. In the case of the DTG thermogram analysis, it was noticed that during the test of each sample there were two peaks in which the rate of sample weight loss increased. The first one occurred at a temperature of approx. 285 °C and was related to the decomposition of linear organic structures forming the polyurethane matrix. The second, more intense peak occurred at a temperature of approx. 375 °C and was associated with further decomposition of carbon chains and aromatic structures derived from the applied isocyanate. The highest weight loss rate for all samples was also at a similar level and ranged from 15.80 to 17.52%/min. A significant change was noted in the case of residues after the test. In the samples modified by halloysite, the residues were always higher than in the case of the REF foam. This was due to the fact that the aluminosilicate filler, which was halloysite, did not undergo thermal decomposition at this temperature and remained after the test.

Based on the conducted thermal tests, it can be noted that modification of flexible PU foams with nano-fillers had no significant influence on the thermal properties of the obtained composites.

## 4. Conclusions

Adding a nano-filler in the form of halloysite to flexible PU foams affected the production process and the physical and mechanical properties of the obtained nanocomposite flexible polyurethane foams. At the synthesis stage, the presence of nano-filler affected the foaming process. It was evidenced by changes in dielectric polarization and temperature as a function of time. A reduction in the maximum temperature from 139 °C for the REF foam to 114 °C for the foams with increasing halloysite content was observed. It is also worth noting that the maximum temperature was reached for a longer time during the nFPUf foaming process compared to the foaming of the REF foam.

Moreover, a significant increase in apparent density from 30.2 kg/m^3^ for the REF foam to 36.8 kg/m^3^ for the H5 foam, a CLD_40_ hardness from 3.1 kPa for the REF foam to 3.7 kPa for the H5 foam, and a favorable reduction in permanent deformation of the modified materials were observed. An analysis of the obtained experimental data confirmed the beneficial effects of the nano-filler on the polyurethane matrix and favorable thermal properties.

The produced foams are characterized by parameters of functional properties that are within the limits required by customers. The presented research results indicate great possibilities of using nano-fillers for the production of flexible polyurethane foam nanocomposites, the synthesis of which is beneficial for both ecological and economic reasons.

## Figures and Tables

**Table 1:** Chemical composition of mined halloysite (in 1 kg of halloysite).

Component	SiO_2_	Al_2_O_3_	Fe_2_O_3_	MgO	CaO	Na_2_O	K_2_O	TiO_2_	SO_3_
Content (%)	46.55	39.50	11.18	0.17	0.66	0.105	0.07	1.69	0.08

**Table 2:** Formulation of reference flexible PU foam.

Foam	Rokopol F3600 (g)	Tegostab BF2370 (g)	DABCO 33LV (g)	DABCO BL-11 (g)	KOSMOS T9	Distilled Water (g)	Ongronat 1080 (g)
REF	100.00	1.00	0.20	0.05	0.20	3.30	41.36

**Table 3:** Detailed description of the FTIR spectra of flexible PU foams.

Wavenumber (cm^−1^)	Bands
3290–3300	Vibrations of nitrogen–hydrogen bonds of urethane and urea groups;
2866–2970	Vibrations of carbon–hydrogen bonds of alkyl groups;
1720–1745	Vibrations of carbonyl groups of urethane and urea groups;
1630–1640	Vibrations of the amide groups;
1598	Vibration of double bonds in the aromatic rings from the isocyanate raw material;
1530–1540; 1449–1453; 1406–1409	Vibration of the aromatic ring structures from diisocyanate raw materials used in the production of flexible PU foams;
1372–1374; 1097–1098	Vibration of carbon–carbon bonds in the PU structure;
1224–1225	Vibration of ether bonds from the polyether polyol;
922–925	Vibration of carbon–hydrogen bonds in aliphatic chains;
670–680	Vibration of carbon–hydrogen bonds in aromatic rings.

**Table 4:** Open-cell content in reference foam and nFPUfs.

Foam Symbol	REF	H1	H2	H3	H4	H5
Content of open cells (%)	94.34 ± 0.87	94.25 ± 0.54	94.28 ± 1.04	94.60 ± 0.36	94.72 ± 1.17	96.49 ± 0.93

**Table 5:** Glass transition temperature and maximum value of tan δ.

Foam Symbol	T_g_ (°C)	tan δ_max_ (-)
REF	−31	0.32
H1	−31	0.29
H2	−33	0.27
H3	−31	0.29
H4	−30	0.31
H5	−31	0.32

**Table 6:** Results of thermal analysis.

Foam Symbol	T_5%_ (°C)	T_10%_ (°C)	T_50%_ (°C)	Highest Weight Loss Rate (%/min)	Residue (%)
REF	255	271	366	15.93	2.53
H1	253	269	359	15.59	5.23
H2	254	270	364	17.52	3.92
H3	251	268	358	15.80	4.68
H4	255	271	361	16.85	6.21
H5	254	270	357	16.80	5.62

## Data Availability

The original contributions presented in the study are included in the article, further inquiries can be directed to the corresponding author.
